# Refractory Ultra-Broadband Perfect Absorber from Visible to Near-Infrared

**DOI:** 10.3390/nano8121038

**Published:** 2018-12-12

**Authors:** Huixuan Gao, Wei Peng, Shuwen Chu, Wenli Cui, Zhi Liu, Li Yu, Zhenguo Jing

**Affiliations:** School of Physics, Dalian University of Technology, 2 Linggong Road, Ganjingzi District, Dalian 116024, China; shark@mail.dlut.edu.cn (H.G.); G21402062@mail.dlut.edu.cn (S.C.); xcuiwenli@163.com (W.C.); liuzhi@mail.dlut.edu.cn (Z.L.); 1050027560@mail.dlut.edu.cn (L.Y.); jingzg@dlut.edu.cn (Z.J.)

**Keywords:** perfect absorber, refractory, ultra-broadband, large incident-angle insensitivity

## Abstract

The spectral range of solar radiation observed on the earth is approximately 295 to 2500 nm. How to widen the absorption band of the plasmonic absorber in this range has become a hot issue in recent years. In this paper, we propose a highly applicable refractory perfect absorber with an elliptical titanium nanodisk array based on a silica–titanium–silica–titanium four-layer structure. Through theoretical design and numerical demonstration, the interaction of surface plasmon resonance with the Fabry–Perot cavity resonance results in high absorption characteristics. Our investigations illustrate that it can achieve ultra-broadband absorption above 90% from a visible 550-nm wavelength to a near-infrared 2200-nm wavelength continuously. In particular, a continuous 712-nm broadband perfect absorption of up to 99% is achieved from wavelengths from 1013 to 1725 nm. The air mass 1.5 solar simulation from a finite-difference time domain demonstrates that this absorber can provide an average absorption rate of 93.26% from wavelengths of 295 to 2500 nm, which can absorb solar radiation efficiently on the earth. Because of the high melting point of Ti material and the symmetrical structure of this device, this perfect absorber has excellent thermal stability, polarization independence, and large incident-angle insensitivity. Hence, it can be used for solar cells, thermal emitters, and infrared detection with further investigation.

## 1. Introduction

Since the concept was first proposed [1], perfect absorbers have attracted widespread attention. In recent years, the development of perfect absorbers has ranged from single-band [2,3] to dual-band [4] and multiband absorptions [5,6]. The regions that achieve perfect absorption include far-infrared [7,8], near-infrared [9], visible light [10,11], and ultraviolet [12,13]. Their performances tend to lead in two directions: achieving a narrow absorption band for sensing performance [14,15], or pursuing a wider absorption band for energy utilization applications such as solar absorbers [16,17].

However, current broadband absorbers can be used with some improvements. First, most traditional broadband absorbers provide broad absorption by combining different sizes of nanostructures in a single cycle, which usually entails complicated production and low efficiency [18,19]. Second, the ultrathin broadband absorbers for the far-infrared band cannot work in the solar frequency band on earth [20,21]. Third, for most proposed broadband absorbers for solar spectral range absorption on earth, their absorption spectrums are not sufficiently wide—the absorption bandwidths above 90% are usually below 1200 nm [22,23,24,25,26]. Therefore, there are urgent practical application needs for ultra-broadband absorbers with high absorption.

In this paper, we design and demonstrate an ultra-broadband perfect absorber of a silica–titanium–silica–titanium (SiO_2_–Ti–SiO_2_–Ti) four-layer structure based on the refractory metal titanium (Ti) with strong plasmonic characteristics. This structure can obtain 1650-nm continuous high absorption from wavelengths of 550 to 2200 nm, with an absorption rate above 90% and an absorption bandwidth much wider than that for previously reported absorbers. This ultra-thin absorber, with a total thickness of 430 nm, can be manufactured using currently available procedures at low cost. It has several advantages over other plasmonic absorbing devices, such as excellent thermal stability, polarization independence, and large incident-angle insensitivity.

## 2. Physical Modeling and Ultra-Broadband Absorption

**Figure 1:**
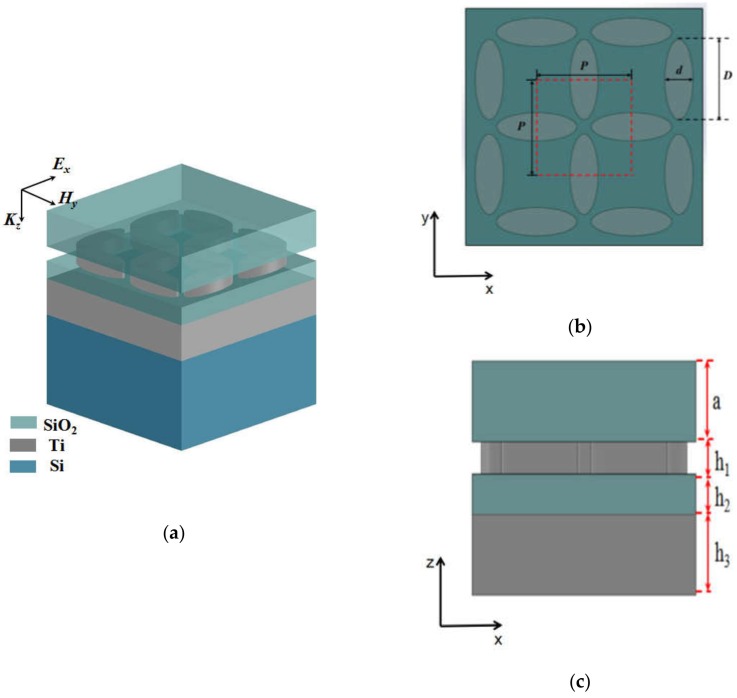
Ultra-broadband perfect absorber. (**a**) Three-dimensional view of the ultra-broadband perfect absorber. (**b**) Top view; the dotted red line represents a full unit cycle in simulation. (**c**) Side view.

We propose an ultra-broadband perfect absorber for visible to near-infrared regions as schematically illustrated in Figure 1. As shown in Figure 1a, the ultra-broadband perfect absorber consists of an SiO_2_ anti-reflection layer combined with a sandwich-structured Ti–SiO_2_–Ti conventional metal–medium–metal perfect absorber structure. The first layer of Ti is composed of an array of ellipses arranged symmetrically. The second layer of Ti is a thick Ti plate, making the transmission of the structure equal to zero. The sandwich layer of the structure is a SiO_2_ layer. The top view of the ultra-broadband perfect absorber is shown in Figure 1b; the dotted red line represents a full unit cell consisting of four semi-elliptical symmetric arrangements, the length in the *x* and *y* directions being *P* = 400 nm. Each elliptical nanodisk has a long-axis length of *D* = 340 nm and a short-axis length of *d* = 120 nm. The side view of the ultra-broadband perfect absorber in Figure 1c shows the thickness of the four layers of SiO_2_–Ti–SiO_2_–Ti, respectively *a* = 150 nm, *h*_1_ = 60 nm, *h*_2_ = 70 nm, and *h*_3_ = 150 nm. Finally, Si is used as a substrate for physical support, which has no effect on absorption.

**Figure 2:**
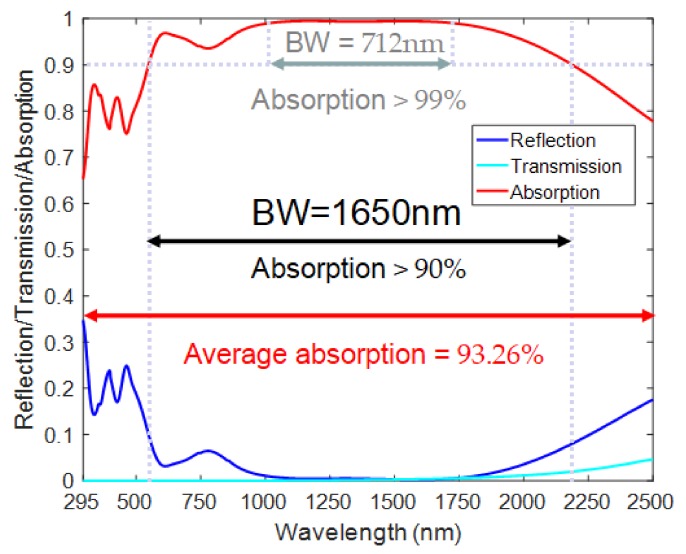
Normalized reflection, transmission, and absorption spectrum under normal incident light. The thickness of the four layers of SiO_2_–Ti–SiO_2_–Ti, respectively are *a* = 150 nm, *h*_1_ = 60 nm, *h*_2_ = 70 nm, and *h*_3_ = 150 nm. The elliptical nanodisk has a long-axis length of *D* = 340 nm and a short-axis length of *d* = 120 nm. The length in the *x* and *y* directions is *P* = 400 nm.

We used a finite-difference time-domain (FDTD) algorithm to simulate the ultra-broadband perfect absorber. To obtain high precision in the simulation, we could only calculate the optical phenomenon of a single periodic array. In the FDTD algorithm, SiO_2_ was selected as a dielectric layer with refractive index 1.45, and the dielectric permittivity of Ti was determined by using experimental data by Palik [27]. We chose a plane wave with wavelengths from 295 to 2500 nm incident on the structure’s surface and a polarized electric field along the *x*-axis. We set the *x* and *y* directions to periodic boundary conditions, and set the *z* direction (incident direction of the light) to a perfect matching layer (PML) boundary condition. The mesh precision was set to 4 nm in the *x* and *y* directions and 2 nm in the *z* direction. As shown in Figure 2, we obtained the normalized reflection, transmission, and absorption spectra of the ultra-broadband perfect absorber under normal incidence. The absorber could provide continuous 1650-nm broadband absorption from wavelengths of 550 to 2200 nm with an absorption rate above 90%. In particular, a continuous 712-nm broadband perfect absorption of up to 99% was achieved from wavelengths of 1013 to 1725 nm.

## 3. Extraordinary Optical Phenomena of Ultra-Broadband Perfect Absorber

### 3.1. Polarization Independence and Large Incident-Angle Insensitivity

**Figure 3:**
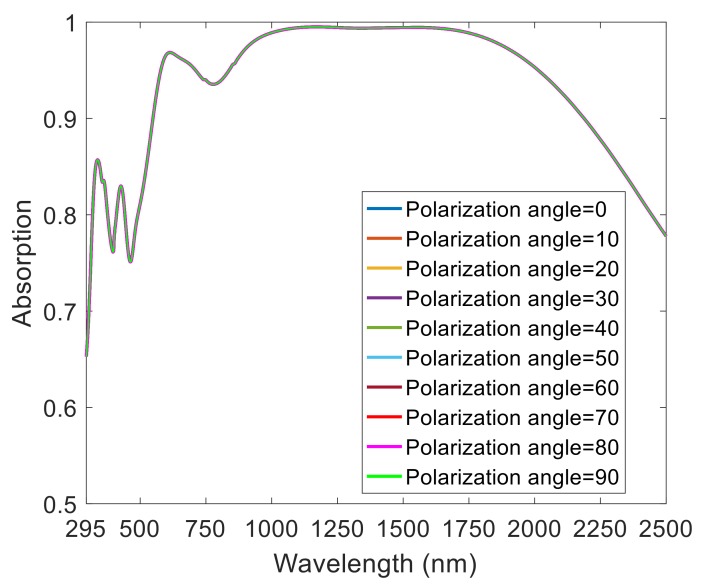
Normalized absorption spectrum of absorber with a polarization angle increasing from 0 to 90° (TE polarization to TM polarization) at normal incidence.

To be applied in a wide range of natural environments, perfect absorbers must have polarization independence and a large angle of incidence insensitivity. As shown in Figure 3, we simulated the normalized absorption spectrum of absorbers with a polarization angle increasing from 0 to 90° (TE polarization to TM polarization) at normal incidence. As the polarization angle increased, the absorption spectrum remained unchanged. This shows that this ultra-broadband perfect absorber is insensitive to polarization, which is caused by the semi-elliptical symmetrical arrangement per period. 

**Figure 4:**
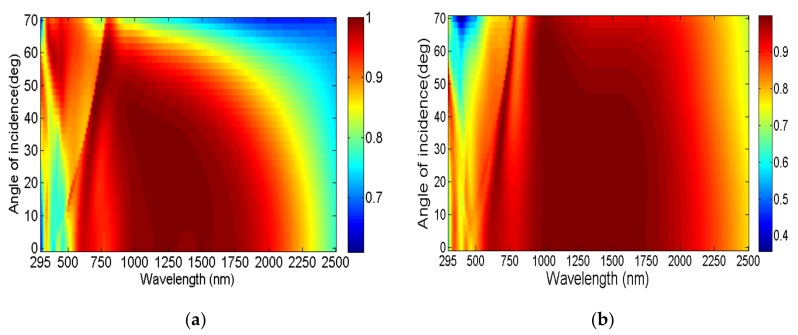
Absorption spectrum under oblique incidence at (**a**) TE polarization and (**b**) TM polarization.

Next, we performed the absorption spectrum simulation under oblique incidence (zero degree of incident-angle mean along the normal to the surface), as illustrated in Figure 4. Figure 4a,b shows the absorption spectrum under oblique incidence at TE polarization and TM polarization, respectively. The ultra-broadband perfect absorber could maintain continuous high absorption over 90% under 50° oblique incidence from wavelengths of 550 to 2000 nm at TE polarization, as shown in Figure 4a. More strikingly, we can see the absorption under oblique incidence with TM polarization from Figure 4b. When the incident angle reached 70°; the absorber could still obtain continuous near-perfect absorption from 750 to 2000 nm. This fully demonstrates that our ultra-broadband absorbers have excellent large incident-angle insensitivity, fundamentally laying the foundation for their superior application.

### 3.2. Influence of Structural Parameters on Absorber Performance

**Figure 5:**
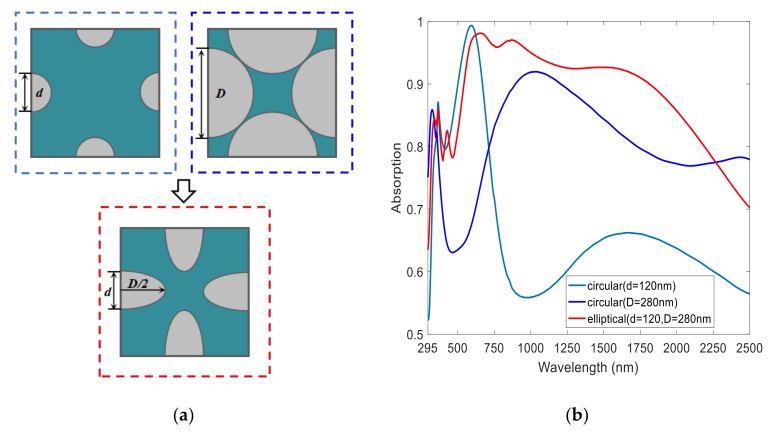
Comparison of the performance of the absorbers under different array patterns. (**a**) Sketch map of circular disk arrays and elliptical disk array. (**b**) Normalized absorption spectra of circular disk arrays and elliptical disk array absorbers.

We compared the absorber performances under different array patterns with all the other parameters unchanged. Figure 5 shows a normalized absorption spectrum of circular disk arrays and elliptical disk array absorbers. It can be seen that the absorption bands of circular disk absorbers varied with the diameter. When the circular disk array had a small diameter of 120 nm, the absorption band was located in a short-wavelength region; when the circular disk array had a large diameter of 280 nm, the absorption band was located in a long-wavelength region. Broadband high absorption was not available in either case. However, when we selected an elliptical disk array (with a long-axis length of 280 nm and a short-axis length of 120 nm), the absorber could achieve continuous broadband absorption from short-wavelength to long-wavelength regions. Therefore, we suspect that the absorption in the short-wavelength region is related to the short axis of the elliptical disks, and the absorption in the long-wavelength region is determined by the long axis of the elliptical disks.

**Figure 6:**
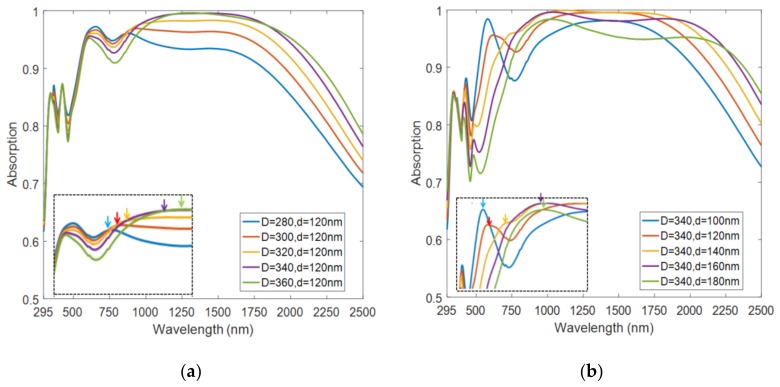
Normalized absorption spectra under different parameters. (**a**) Absorption spectrum of a constant short-axis length of the elliptical nanodisk with an increased length of the long axis. (**b**) Absorption spectra of a constant long-axis length of the elliptical nanodisk with an increased length of the short axis.

To verify this conjecture, we further investigated the absorption spectrum change by separately modifying the lengths of both axes, as shown in Figure 6. When we kept the short-axis length of the elliptical nanodisk constant and increased the length of the long axis, we found that the short-wavelength absorption band of the absorber was almost unchanged, and the long-wavelength absorption band was red-shifted as shown in Figure 6a (inset). Figure 6b illustrates that when we kept the long-axis length of the elliptical nanodisk unchanged and increased the length of the short axis, the short-wavelength absorption band was red-shifted. Additionally, an absorption dip appeared between the long-wavelength and short-wavelength absorption bands when the long-axis dimension of the ellipse was much larger than the short-axis dimension (*D* = 340 nm, *d* = 100 nm). The large difference in size between the long and short axes led to the separation of two absorption peaks of short- and long-wavelength bands. Therefore, we conclude that in this perfect absorber, the short-wavelength band absorption is related to the short axis of the Ti elliptical disk, while the long-wavelength band absorption is affected by the long axis of the Ti elliptical disk.

**Figure 7:**
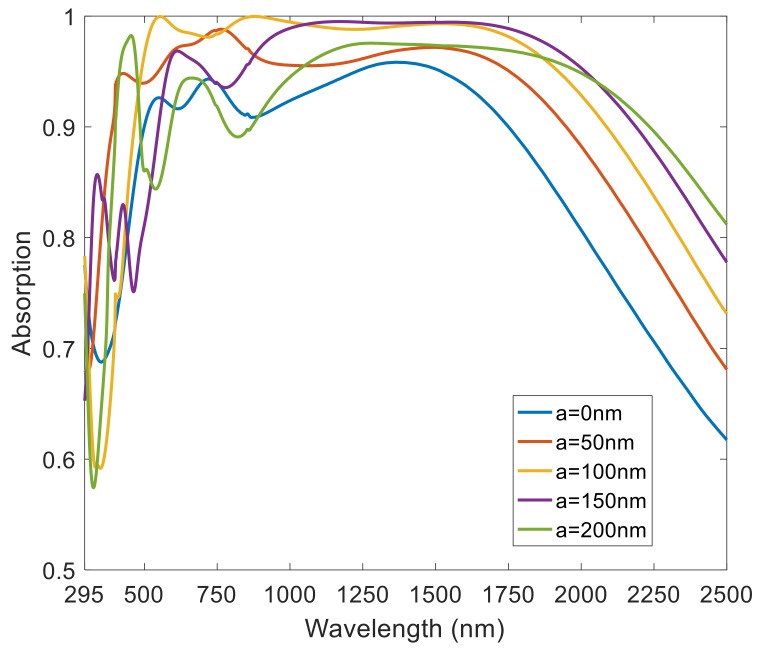
Normalized absorption spectra under different thicknesses of the anti-reflection layer.

Next, we analyzed the effect of the antireflection layer’s thickness on the absorption properties. As shown in Figure 7, the presence or absence of the antireflection layer influenced the ultra-broadband perfect absorber greatly. We chose SiO_2_ as the antireflection layer because it is a transmittive material from deep ultraviolet to mid-infrared. From the absorption spectrum, the absorption rate of the absorber increased at first and then decreased with the increase of the thickness of the SiO_2_ layer. This can be interpreted as follows: When the anti-reflection layer thickness increases, the absorption rate of this device will gradually increase. However, a thick SiO_2_ layer will block the incidence of electromagnetic waves and lead to a decrease of the absorption rate.

In addition, this ultra-broadband perfect absorber has excellent thermal stability, because the Ti melting point can reach up to 1668 °C, which is much higher than that of gold (i.e., 1064 °C). It has been verified that Ti materials and their composite micro/nano devices are more stable in harsh environments (e.g., high-temperature and laser environments) than other materials [10]. Another advantage of Ti metal as a plasmonic material is its abundant reserves—it ranks tenth among all element reserves on the earth, which is 61 times that of copper.

### 3.3. Super Capture Capability under Air Mass (AM) 1.5 Solar Spectrum

**Figure 8:**
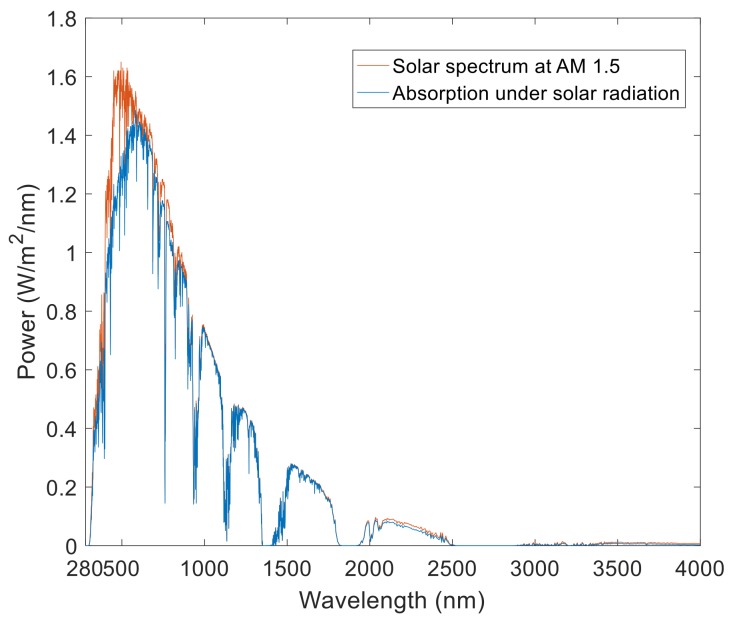
Solar spectrum at air mass (AM) 1.5 and absorption spectrum of the ultra-broadband perfect absorber.

In FDTD, we used air mass (AM) 1.5 to simulate the absorption of the ultra-broadband perfect absorber under an ideal solar light source. As shown in Figure 8, the simulated absorption was highly coincident with the solar spectrum. The average absorption rate of solar energy from wavelengths of 280 to 4000 nm by the ultra-broadband perfect absorber was 88.16%, which shows a supercapturing ability for sunlight. Moreover, since the wavelength range of sunlight reaching the earth is from 295 to 2500 nm, selecting the solar spectrum at this band, the average absorption of the ultra-broadband perfect absorber was 93.26%, which shows a very strong solar absorption rate on earth. These technical characteristics make this absorber a superior device for many applications in the natural environment, such as solar cells, solar collectors, and thermal devices.

## 4. The Physical Origin and Simple Fabrication Method of the Ultra-Broadband Absorber

**Figure 9:**
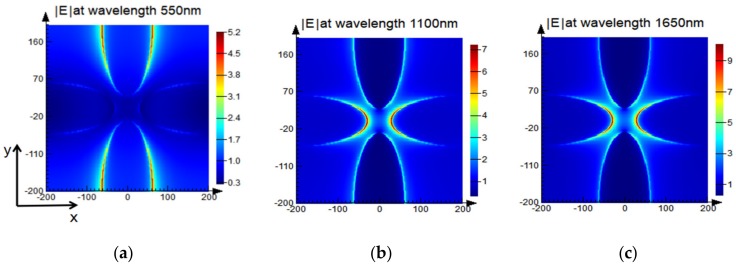

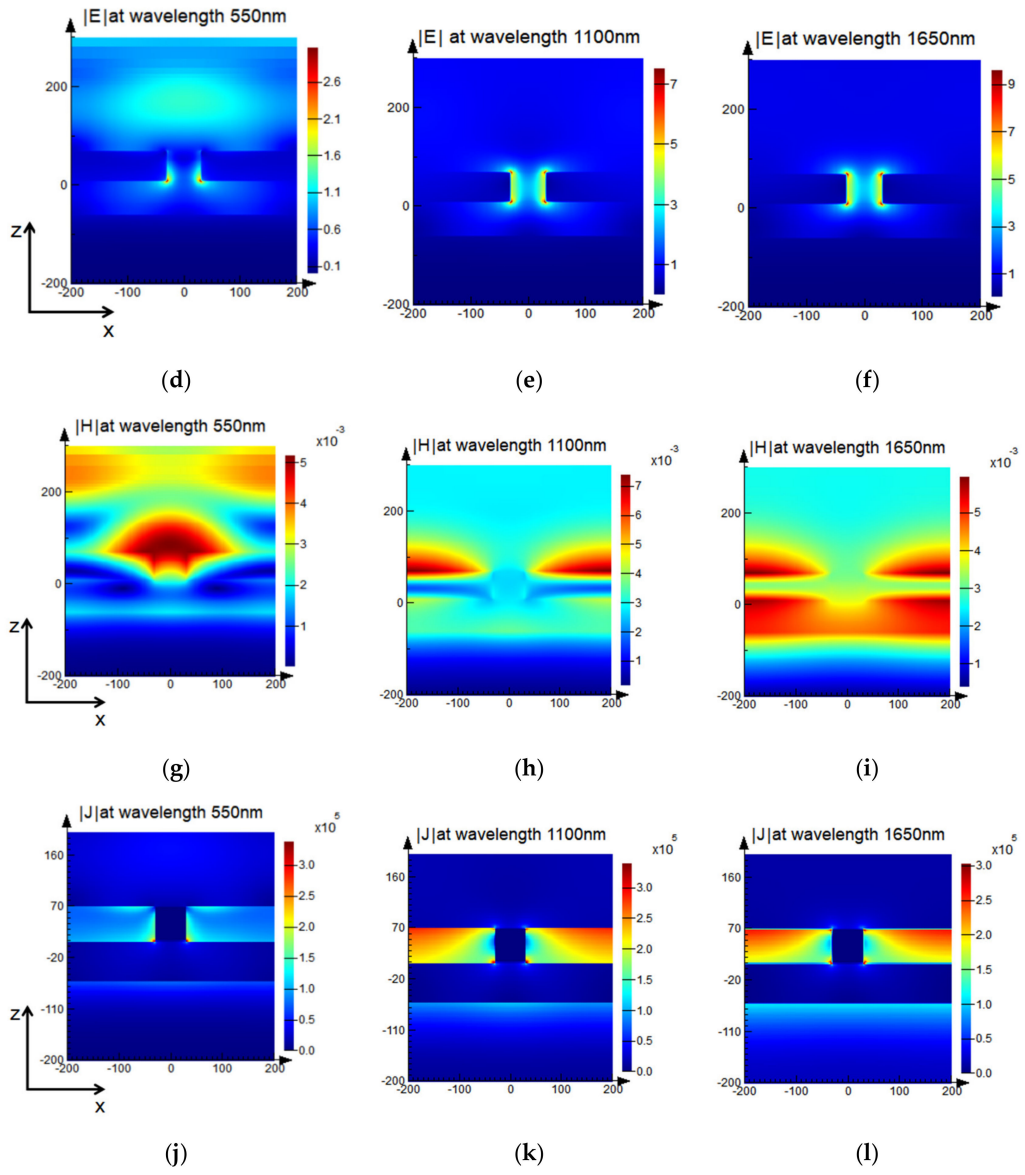
Electromagnetic field intensity and current distribution of the TM-polarized light with a normal incidence at various resonant wavelengths (i.e., 550, 1100, and 1650 nm). (**a**–**c**) Electric field intensity distribution at the interface between the SiO_2_ antireflection layer and the Ti elliptical nanodisk. (**d**–**f**) Electric field intensity distribution in the *x*–*z* plane when *y* = 0. (**g**–**i**) Magnetic field intensity distribution in the *x*–*z* plane when *y* = 0. (**j**–**l**) Current distribution in the *x*–*z* plane when *y* = 0.

The physical origin of the ultra-broadband perfect absorber is the interaction of surface plasmon resonance (propagating surface plasmon and localized surface plasmon) with the Fabry–Perot cavity resonance. To illustrate this principle, we calculated the electromagnetic field intensity and current distribution of the TM-polarized light with a normal incidence at various resonant wavelengths (i.e., 550, 1100, and 1650 nm), with the results shown in Figure 9. First, it can be seen from Figure 9a–f that surface plasmons are excited at the Ti elliptical nanodisk in each period, as manifested by the fact that the electric field is localized at the tip and induces absorption. The surface plasmon resonance at the resonance wavelength of 550 nm occurred mainly between the short axes of the elliptical disk, while the surface plasmon resonance at the resonance wavelength of 1650 nm occurred mainly between the long axes of the elliptical disk. This is the same as the results shown in Figure 6, verifying our previous analysis. Second, we can see that there was a significant difference in the magnetic field distribution from Figure 9g–i. The magnetic field was localized in the gap between the Ti elliptical nanodisks at wavelength 550 nm, indicating the local surface plasmon (LSP) resonance control structure absorption. However, the magnetic field was mainly distributed in the SiO_2_ dielectric layer and the SiO_2_ antireflection layer at wavelength 1650 nm, indicating that the propagation surface plasmon (PSP) resonance plays a major role in the absorption. In addition, the Ti–SiO_2_–Ti three-layer structure constitutes a Fabry–Perot cavity, and Fabry–Perot (FP) resonance is also a cause of structural absorption. Therefore, the combined action of PSP, LSP, and FP-like resonance resulted in a high absorption rate at the resonance wavelength of 1100 nm. Finally, as shown in Figure 9j–l, the current was distributed mainly on the Ti elliptical nanodisk and the top of the thick Ti layer, indicating that broadband absorption is also derived from the intrinsic loss of the metal Ti. In summary, the ultra-broadband perfect absorption of the device is caused by the interaction of PSP, LSP, and FP-like cavity resonance.

**Figure 10:**
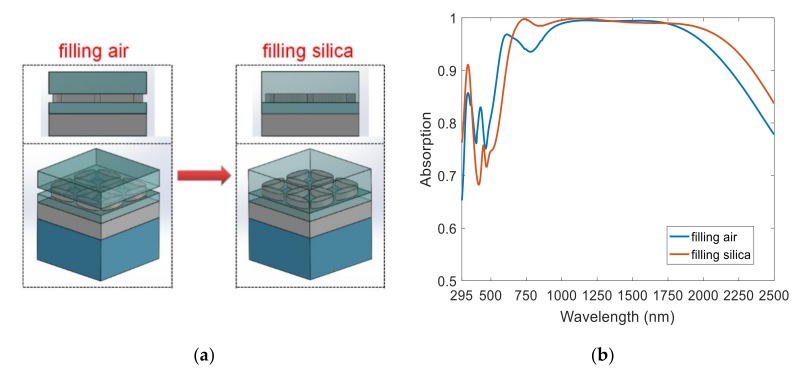
Filling air and filling silica absorber: (**a**) schematic diagram, and (**b**) normalized absorption spectra.

In order to improve the applicability of this ultra-broadband perfect absorber, we propose a simple, low-cost fabrication method. Due to environmental filling, the content between the two layers of SiO_2_ is air of the ultra-broadband perfect absorber, apart from the Ti elliptical disk. Since this is not conducive to simple fabrication, we replaced the environmental filling with low-refractive-index SiO_2_, as shown in Figure 10a. Our simulations demonstrated that filling SiO_2_ still maintained the ultra-broadband absorption compared to the air-filled absorber, with only a slight red shift in the absorption band, as shown in Figure 10b. The fabrication procedures are as follows: first, we deposit 150-nm-thick Ti and 70-nm-thick SiO_2_ on the surface of the Si substrate using magnetic sputtering; second, we spin-coat 150-nm-thick polymethyl methacrylate (PMMA) photoresist on the top surface of the substrate; third, we form the inverse structure of the target structure using electron-beam exposure and development; fourth, we develop an electron-beam deposition of a 60-nm-thick Ti metal layer, stripping it and forming a metal Ti nanostructure; and finally, we deposit 210-nm-thick SiO_2_ on the structure surface and planarize the surface of the nanostructure. Work is ongoing to develop this structure for experimentation and testing and eventual application, which we will report on separately upon further investigation.

## 5. Conclusions

We theoretically designed and mathematically demonstrated an ultra-broadband perfect absorber that operates continuously from visible to near-infrared regions. Based on a SiO_2_–Ti–SiO_2_–Ti four-layer structure, the interaction of surface plasmon resonance with the Fabry–Perot cavity resonance resulted in perfect absorption of this perfect absorber. It could be applied to the entire solar band from 295 to 2500 nm on the earth, enabling continuous near-perfect absorption in the 550–2200 nm band, where the absorption rate was maintained above 90%. In particular, its average absorption of all sunlight on the earth reached 93.26%, which indicates a super-strong solar-light-capturing ability in the natural environment. This broadband perfect absorber is made of refractory metal Ti with excellent thermal stability. The independence of polarization and insensitivity of large incident angles make this perfect absorber an ideal device with significant potential for energy utilization applications such as solar cells and solar collectors.
